# Can somatostatin control acute bleeding from oesophageal varices in *Schistosoma mansoni *patients?[ISRCTN63456799]

**DOI:** 10.1186/1471-2334-4-58

**Published:** 2004-12-13

**Authors:** Shyama Chatterjee, Eric Van Marck

**Affiliations:** 1 Laboratory of Pathology, Faculty of Medicine, University of Antwerp, Universiteitsplein-1, 2610 Antwerp, Belgium

## Abstract

**Background:**

Management of patients with bleeding oesophageal varices comprises of mainly diagnostic endoscopy, sclerotherapy and band ligation. One of the major problems to do any of the above is the active bleeding which makes any intervention difficult. The neuropeptide hormone somatostatin administered exogenously has caused a reduction in portal hypertension and variceal bleeding in patients suffering from liver cirrhosis. We believe that the symptomatic use of somatostatin for variceal bleeding in *Schistosoma mansoni *infected subjects can reduce bleeding, thereby alleviating the pathology caused by schistosomiasis.

**Methods/design:**

We herein present a study protocol for establishing this neuropeptide as a potential therapeutic agent in schistosomiasis. Adolescent subjects, age range varying from 12–17 years will be selected, based on several inclusion criteria, most important being infection with *Schistosoma mansoni *with bleeding from oesophageal varices in the last 24 hours. One group of schistosomiasis patients will be treated with somatostatin and praziquantel, the other with propanolol and praziquantel. Survival graphs will be set up to correlate somatostatin administration with survival time. A two part questionnaire will be set up to control treatment outcomes. The pre-treatment part of the clinical questionnaire will identify inclusion criteria questions, the post-treatment part of the questionnaire will identify treatment outcomes.

**Discussion:**

We expect that the administration of somatostatin as a bolus followed by a 24 hour long infusion, will stop bleeding immediately, delay rebleeding as compared to the control study group and delay mortality in the somatostatin treated subjects.

## Background

Complications due to severe schistosomiasis include fibrosis, hepatomegaly, splenomegaly, haematemesis, varices, portal hypertension, ascites formation and death [1]. Complications resulting from hepatic fibrosis (such as portal hypertension and variceal bleeding) are the principal cause of death in *S. mansoni *infected patients. In such patients portal hypertension leads to the formation of portal-systemic collaterals, specifically gastro-oesophageal collaterals (varices). Bleeding of these oesophageal varices can occur depending on the severity of fibrosis, and can be fatal. Praziquantel, the most commonly used anti-schistosomal drug, is effective against the worm stages of the parasite, but has no activity against the pathology (fibrosis) caused by the egg stages or the variceal bleeding that can be fatal in its outcome. These observations have stressed the need to combine praziquantel treatment with symptomatic treatment like with somatostatin.

Somatostatin is emerging as the ideal vasoactive drug for the control of variceal bleeding, and is as effective as sclerotherapy [2-4]. In a recent clinical trial, cirrhotic patients with acute bleeding of oesophageal varices were treated with infusion followed by bolus injections of somatostatin just before sclerotherapy. Results showed fewer treatment failures, fewer deaths or use of rescue therapy, reduced blood transfusion and less frequent, active bleeding. This drug also prevents recurrence and is free from any major side effects even when administered over prolonged periods of time.

We have studied the potential role of somatostatin in modulating *Schistosoma *caused morbidity. In endemic regions, at any given time, only a fraction of infected patients develop severe hepatic fibrosis. There may be a direct correlation between the development of severe fibrosis and the inability to generate required somatostatin levels. Our ongoing research at the Laboratory of Pathology give evidence to this fact, since somatostatin levels in Senegalese patients with severe morbidity (haematemesis, portal hypertension, variceal bleeding, ascites, fibrosis) are significantly lower than that in patients with no severe morbidity [5-10]. This effect indicates that somatostatin could be administered exogenously for therapeutic purposes in chronic schistosomiasis patients.

Complications linked to hepato-intestinal schistosomiasis are increasing in the area of Richard-Toll, Northern Senegal. These cases are being identified at the local health centre. However, considering the high prevalence of schistosomiasis in this region, it is likely that the number of severe cases is much higher. Clear guidelines for the management of such severe complications and criteria for referral and surgery are required in this region. The establishment of an algorithm on how to treat these patients and create the appropriate infrastructure is urgently needed. The priority is how to take care of these patients and more so what is the best way of doing this under local conditions. One of the first steps is to critically evaluate the Niamey-Belo-Horizonte ultrasound methodology in patients with severe periportal fibrosis. Efficient management of bleeding varices in the afflicted patients is imperative.

Given the background that somatostatin is an ideal vasoactive drug in the field of liver pathology, it is our opinion that somatostatin will be more efficacious and safe as compared to currently used beta blocker drugs like propanolol, in the control of acute oesophageal variceal bleeding due to *Schistosoma mansoni *infection. Moreover using this neuropeptide may increase time to failure of drug treatment, decrease incidences of early re-bleeding (day 4, 8) and incidences of death during the follow up period. Decreased frequencies of late rebleeding (days 30, 60, 90) may occur, all indicating the safety of using somatostatin. Praziquantel cover would be given to all study patients.

### Study design

#### 1. Selection of patients

Age and Morbidity criteria – Adolescent subjects, age range varying from 12–17 years will be selected. The inclusion criterion will be schistosomiasis patients with bleeding from oesophageal varices in the last 24 hours. A random selection will be made to form two groups, a study group and a control group. Control of active infection will be done by means of CAA-strips on urine or blood. Subjects will be asked to fill in an informed consent form and the pre-treatment part of a questionnaire.

The inclusion criteria will be established fibrosis due to schistosomiasis of clinical history, physical examination and laboratory findings (and an examination compatible with the presence of portal hypertension due to fibrosis). Clinically active upper gastrointestinal bleeding (haematemesis of fresh or semi fresh blood and/or melena and/or haematochezia) with or without haemodynamic instability (systolic blood pressure < 80 mm Hg and heart rate > 120 bpm) will be selected. Subjects must be male or non-pregnant, non-lactating female subjects. Females of childbearing potential will have to utilize contraception for the duration of the study. Written or verbal documented informed consent will be needed from all subjects.

Exclusion criteria will include participation by subjects in another investigational study within the last 14 days. Subjects may not undergo treatment with endotherapy, i.e. band ligation, sclerotherapy or other (balloon tamponade). Treatment with somatostatin, vasopressin or their analogues will also be a exclusion criteria. Subjects with end stage liver disease with hepatorenal syndrome, diffuse hepatocellular carcinoma, patent porto-systemic shunts, known diagnosis of non-fibrotic portal hypertension, severe cardiovascular diseases, i.e. acute myocardial infarction and heart failure will be excluded. Concurrent use of metoclopramide is also not advised.

Conduct of trial – Active bleeding episode (haematemesis, haematochezia, melena) from a potential variceal source should be confimed by a medical team (the ER physician, the ICU physician, the investigator). Patients may be outpatients or already hospitalised patients. Patients will be randomised to either arm in a sequential manner. Randomisation, and the start of study drug infusion if in adjunctive therapy arm, should be accomplished as soon as possible following identification of a patient qualifying for the study and following the conduct of pre-randomisation study procedures.

Sample Size Criteria – Assuming a 80% chance of finding a significant difference <0.05 between the two study cohorts, the following statistics were established:

(A) If 99% of the untreated subjects and –

10% of somatostatin treated subjects bled -5 volunteers per group were sufficient;

20% treated subjects bled – 7 volunteers per group were required;

30% treated subjects bled-9 volunteers per group were needed.

(B) If 90% of untreated subjects bled, and –

10% of somatostatin treated group bled – 7 volunteers in each cohort were sufficient;

20% of somatostatin treated group bled – 9 volunteers in each group were required;

30% of somatostatin treated group bled – 10 volunteers in each group were needed.

(C) If 70% of untreated subjects bled, and:

10% of somatostatin treated group bled – 12 volunteers per group were sufficient;

20% of somatostatin treated group bled – 18 volunteers per group were required;

30% of somatostatin treated group bled – 28 volunteers per group were needed.

In cirrhotic patients with bleeding oesophageal varices somatostatin administration controls bleeding in more than 80% of the treated patients. Based on this report, we propose to start this pilot study with 10 subjects/group.

#### 2. Treatment

Two groups of 10 schistosomiasis patients each will be identified. Group (1) will be treated with Somatostatin (3.5 μg/kg/hour; single bolus and i.v. infusion for 24 hours) + Praziquantel (40 mg/kg). For somatostatin treatment the i.v. infusion will be started first; 3 mg somatostatin will be dissolved in the 1 ml of saline provided. This solution will be added to the saline transfusion unit and administered to the patient for the next 12 hours. Once finished the second packet of 3 mg somatostatin will be used similarly for a second saline transfusion unit for the remaining 12 hours. The bolus dose of 250 μg will be dissolved in the 1 ml of saline provided and administered over 90 seconds soon after the start of the i.v. infusion.

Group (2) will be treated with Propanolol + Praziquantel.

#### 3. Data analysis

Survival graphs will be set up to correlate somatostatin administration with survival time.

The primary efficacy variable is the number of patients meeting the failure of therapy definition during the infusion period. Failure criteria are defined as death during infusion, persistence of active bleeding (The haemodynamic instability criteria points to the inability to achieve and maintain a systolic blood pressure of 80 mm Hg OR presence of a 20 mmHg drop in systolic blood pressure from the highest post resuscitation value AND achieving a heart rate of 120 bpm OR a 20 bpm increase from highest post resuscitation value OR Inability to achieve and maintain a Hct of – 27% of Hb of – 9 g/dl despite blood transfusion of 2 units or more.

The clinical criteria of active bleeding include hematemesis (fresh or semi fresh blood), hematochezia, melena.

#### 4. Control points

A two part questionnaire will be set up to control treatment outcomes. The pre-treatment part of the clinical questionnaire will identify inclusion criteria questions:

(1) When was the last haematemesis incidence?

(2) When did the present incident start?

The post-treatment part of the questionnaire will identify treatment outcomes. The following questions will be answered:

(1) What was the reaction to somatostatin infusion?

(2) When did bleeding stop after somatostatin infusion?

(3) GI disturbances: Is there abdominal pain, nausea, diarrhea, after somatostatin infusion?

(4) Control of early rebleeding: Is there early rebleeding in the 8 days following somatostatin administration?

(5) Control of late rebleeding: When is the next rebleeding incident? Subjects will be followed up on a monthly basis, by home visits.

(6) Control of mortality: Is there any difference in mortality time between the two groups.

(7) Control of fibrosis: Does ultrasonography detect anti-fibrotic effect of somatostatin after treatment?

## Discussion

It is expected that the administration of somatostatin as a bolus followed by a 24 hour long infusion, will stop bleeding immediately, delay rebleeding as compared to the control study group and delay mortality in the somatostatin treated subjects. Our protocol that is based on a pilot study will help to establish the importance of somatostatin in schistosomiasis.

## Competing interests

The author(s) declare that they have no competing interests.

## Authors' contributions

Both authors participated in the design of the study.

## Pre-publication history

The pre-publication history for this paper can be accessed here:


